# Assessing the Concordance of Clinical and Pathological Diagnoses in Basal Cell Carcinoma Among the Iranian Population: A Cross‐Sectional Analysis of 229 Cases

**DOI:** 10.1002/cnr2.70040

**Published:** 2024-10-28

**Authors:** Fatemeh Farshad, Elham Behrangi, Alireza Jafarzadeh, Masoumeh Roohaninasab, Nasrin Shayanfar, Zeinab Aryanian, Parvaneh Hatami, Azadeh Goodarzi

**Affiliations:** ^1^ School of Medicine Iran University of Medical Sciences Tehran Iran; ^2^ Department of Dermatology, Rasool Akram Medical Complex Clinical Research Development Center (RCRDC), School of Medicine Iran University of Medical Sciences Tehran Iran; ^3^ Department of Dermatopathology, Rasool Akram Medical Complex Iran University of Medical Sciences Tehran Iran; ^4^ Autoimmune Bullous Diseases Research Center Tehran University of Medical Sciences Tehran Iran; ^5^ Department of Dermatology Babol University of Medical Sciences Babol Iran

**Keywords:** clinical assessment, diagnostic accuracy, histopathological findings, referral patterns, skin cancer education

## Abstract

**Background:**

Nonmelanoma skin cancer (NMSC) is the most prevalent malignancy globally, with basal cell carcinoma (BCC) being the most common type.

**Aims:**

This study aims to evaluate the concordance between clinical and pathological diagnoses of BCC, emphasizing the importance of early detection.

**Methods and Results:**

In this cross‐sectional study, we conducted a retrospective review of clinical and pathological records for 229 patients diagnosed with BCC between 2020 and 2024. The analysis focused on gender, age, lesion location, and diagnostic accuracy. Among the 229 patients, 193 were men (84.3%), and 131 (57.2%) had recorded clinical diagnoses. The mean age of diagnosed patients was 67.72 years. Lesions were primarily located on the scalp (29.5%), face (26.4%), and nose (13.9%). Of the pathological evaluations, 184 cases (80.3%) confirmed BCC, while 45 cases had alternative diagnoses. Notably, 94.6% of clinically diagnosed patients were suspected to have BCC by their physicians. A significant portion of cases (42%) lacked prior clinical diagnoses, reflecting a potential gap in education among nondermatologists regarding BCC recognition.

**Conclusion:**

The study found high concordance between clinical and pathological diagnoses of BCC, underscoring the need for improved clinical assessment skills among healthcare providers. Collaboration with dermatologists is essential for accurate diagnosis and improved patient outcomes. Enhanced training in recognizing BCC symptoms is recommended to address the identified gaps in clinical suspicion.

**Abbreviations**
BCC: Basal cell carcinoma
IT: Information technology
NMSC: Nonmelanoma skin cancer
WHO: World Health Organization

## 1 Introduction

Basal cell carcinoma (BCC) is one of the most prevalent malignant tumors worldwide, and its incidence has significantly increased in recent years [1, 2]. This slow‐developing cancer is characterized by its localized invasive nature and an extremely low tendency for metastasis. BCC primarily affects sun‐exposed skin areas in older adults, with a particular predilection for the maxillofacial region [1, 3]. Skin cancers, including BCC, have a considerable negative impact on patients' quality of life, contributing to a growing global health concern. The World Health Organization (WHO) estimates that 2–3 million cases of nonmelanoma skin cancer (NMSC) and approximately 132 000 melanoma cases occur globally each year [4, 5, 6]. Notably, BCC is considered the most prevalent malignancy in humans, with an annual incidence of around 8% reported in the USA as of 2018. Alarmingly, recent studies indicate a rising prevalence of BCC, accompanied by a decreasing age of onset [7, 8].

Multiple risk factors are associated with BCC, with ultraviolet (UV) radiation recognized as the primary cause. Individuals with lighter skin and hair, those who have experienced recent sunburns, or those who work outdoors or have a history of phototherapy are at increased risk [9, 10]. The use of photosensitizing medications, such as tetracyclines and thiazides, and immune suppression also heightens the likelihood of developing BCC. While BCC tumors are rarely metastatic, their local invasion can result in significant tissue damage, underscoring the necessity for prompt and effective diagnosis [11, 12, 13].

Accurate diagnosis of BCC is crucial, as early intervention can prevent the severe consequences associated with advanced tumors. The sensitivity and specificity of visual examinations for BCC detection are reported at 85% and 97.2%, respectively, while dermoscopy enhances diagnostic accuracy with sensitivity and specificity rates of 91% and 95% [14, 15]. Dermoscopy allows for the identification of specific patterns such as the “blue‐white veil,” “arborizing vessels,” and “peripheral ova,” which are instrumental in differentiating BCC subtypes, including nodular, superficial, and morpheaform types [16]. Furthermore, scraping cytology can serve as a beneficial method in outpatient settings, facilitating quicker diagnosis without the scarring risks associated with biopsies [16].

This study aims to evaluate the concordance between clinical and pathological diagnoses of BCC within the authors' affiliated hospital. We will analyze the impact of lesion site and subtype on clinical judgment, explore the correlations between clinicopathological features, and assess the utility of classic dermoscopy in enhancing diagnostic accuracy among various BCC types. Given the high occurrence of BCC, particularly among vulnerable populations such as the elderly and those with specific skin characteristics, this research underscores the critical nature of accurate pathological diagnoses, particularly in the context of evolving BCC subtypes and their clinical implications.

## 2 Materials and Methods

### 2.1 Patients

The study population consisted of patients with a confirmed pathology report for BCC, verified by a board‐certified dermatopathologist. Patients' clinical records were extracted from the hospital's Information Technology (IT) system for the period between 2020 and 2024. Data collected included demographics such as age, gender, and history of radiotherapy. Additionally, the specific subtype of BCC, as confirmed through pathology reports, was documented along with the clinical diagnoses registered by attending physicians. This comprehensive approach ensured that a representative sample of patients was analyzed, providing insights into the demographic characteristics and clinical backgrounds of those affected by BCC.

### 2.2 Assessment Method

To collect relevant data, the study conducted a comprehensive retrospective review of existing medical records and pathology reports. Only those patients with a confirmed diagnosis of BCC were included in the analysis. The assessment process included extraction of demographic information, information related to prior radiation exposure, and detailed information about the subtype of BCC diagnosed. Clinical diagnoses made by healthcare providers, recorded in the patients' charts, were compared to the pathological confirmation. This comparison aimed to evaluate the concordance between clinical assessments and histopathological findings. Furthermore, the study adhered to ethical guidelines, ensuring that all patient information was anonymized to maintain confidentiality throughout the research.

### 2.3 Data Analysis

The extracted data were analyzed using SPSS version 25.0, a statistical software package widely used for data analysis in medical research. Descriptive statistics were calculated to summarize the demographic and clinical characteristics of the patient population. The chi‐square test was applied to evaluate the diagnostic agreement between clinical diagnoses and pathological confirmations. This method was essential for assessing the significance of the relationship between categorical variables in our dataset, particularly in regard to the referral system's diagnostic compatibility. All analyses were conducted anonymously to further protect patient privacy, and a significance level of *p* < 0.05 was considered statistically significant. The results obtained from this analysis were instrumental in understanding the accuracy and reliability of clinical diagnoses relative to pathological findings in the context of BCC.

### 2.4 Ethical Considerations

The participants in this project adhered to all Helsinki ethical principles. This research was approved by the Research Council with the ethics code number IR.IUMS.FMD.REC.1397.249.

## 3 Results

The clinical records of 229 pathologically confirmed patients with BCC lesions were reviewed from 2020 to 2024. Among these patients, 193 were men (84.3%) and 36 were women (15.7%), indicating a significant male predominance in the incidence of BCC (Table 1).

**TABLE 1:** The demographic information of the patients with recorded clinical diagnosis.

Age (mean ± SD), years	67.72 (±10.0)
Gender	Male: 108 (82.4%) Female: 23 (17.6%)
History of radiation	60 (45.8%)
Total	131 (100%)

Out of the 229 patients, 131 (57.2%) had a clinical diagnosis noted (clinical suspicion), while 98 patients (42.79%) did not have any documented clinical diagnosis prior to their referral for pathology assessment.

Among the 131 patients with a clinical diagnosis, the mean age was 67.72 years (±10.00), with 108 (82.4%) being male and 23 (17.6%) female. These data reflect a consistent pattern of gender distribution across age groups.

### 3.1 Lesion Distribution

**FIGURE 1:**
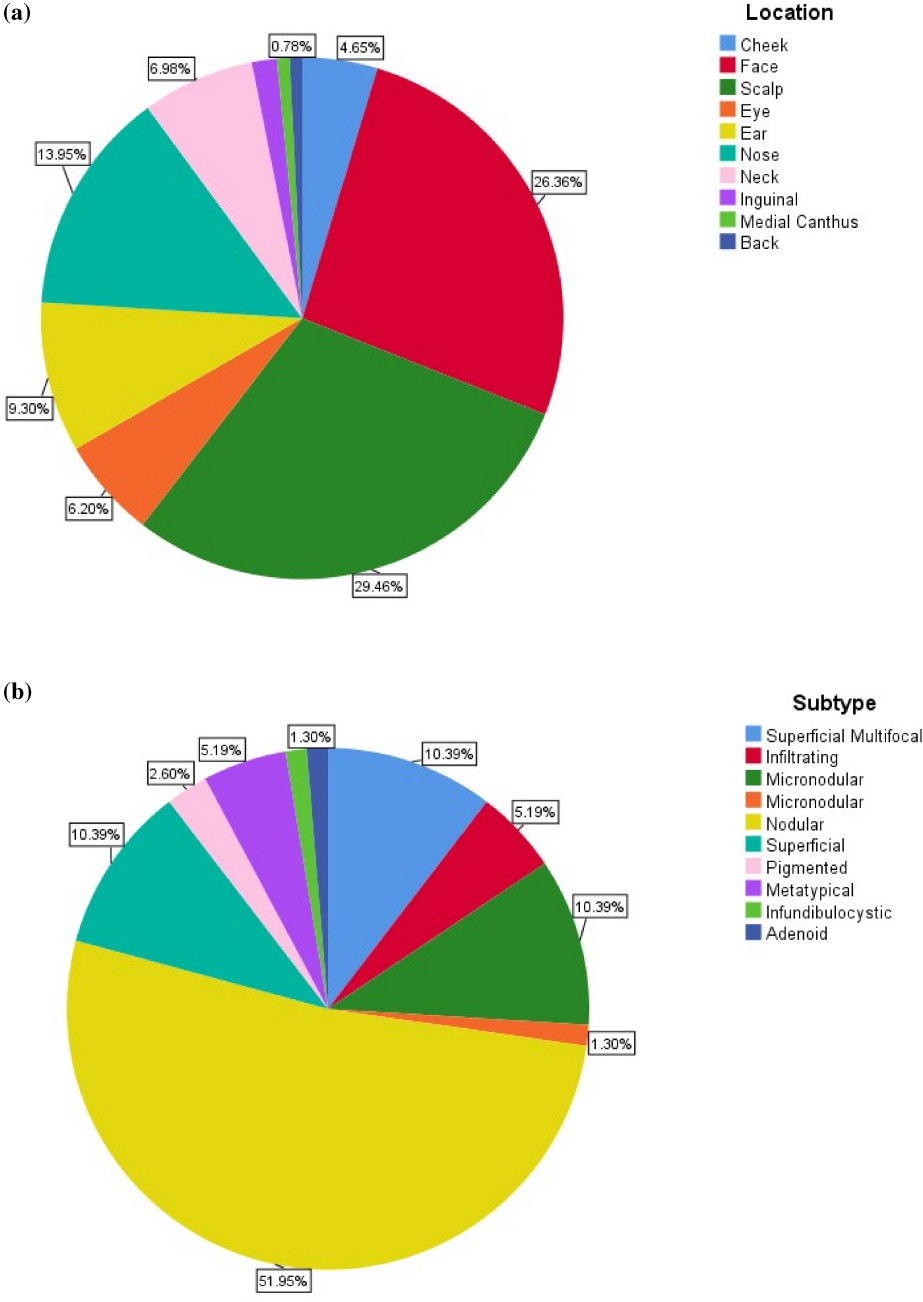
Locations (a) and subtypes (b) of the pathologically confirmed lesions included in the study.

**FIGURE 2:**
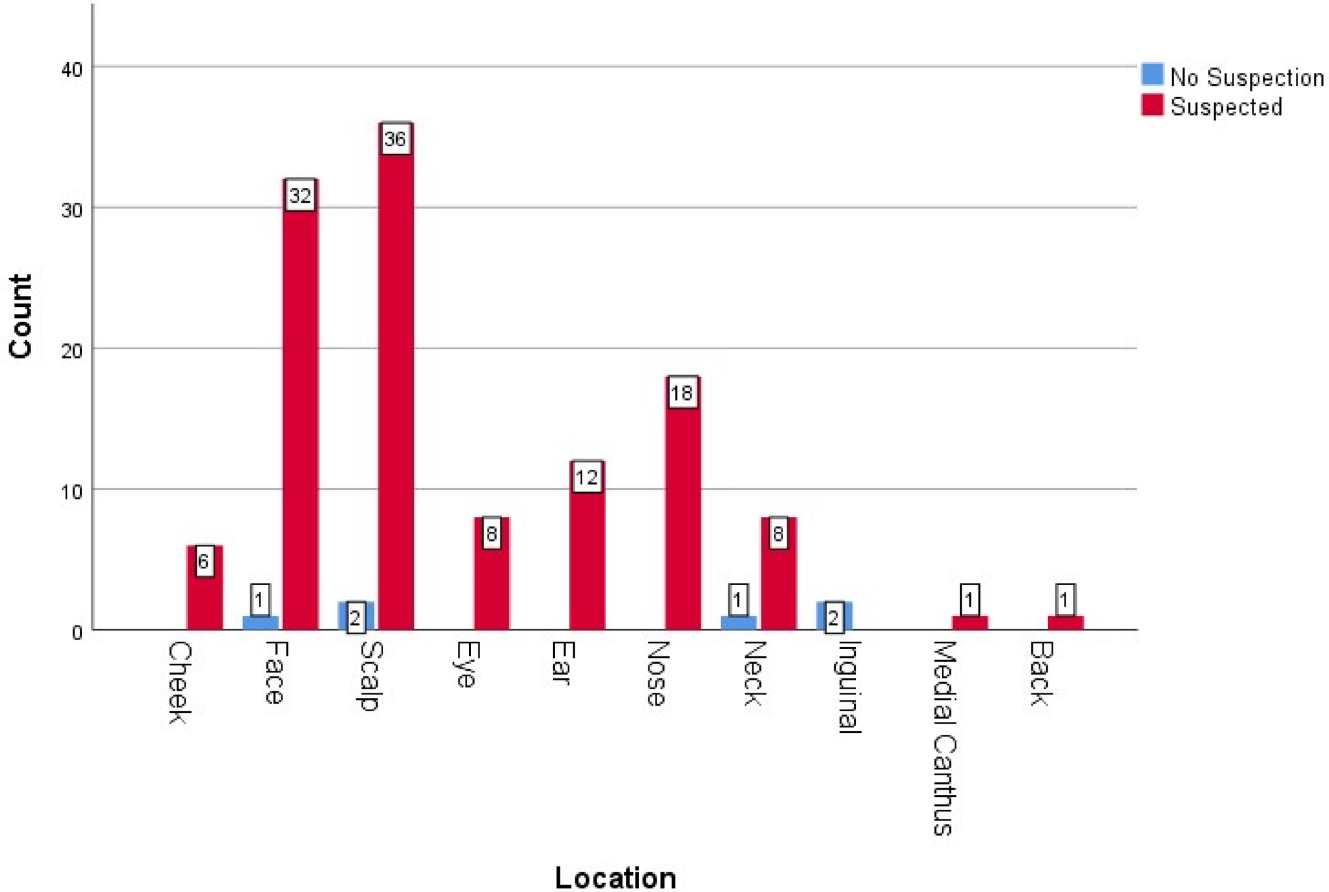
The frequency of suspected and nonsuspected BCC lesion in each location.

A detailed analysis of lesion locations revealed that the scalp was the most affected area, accounting for 29.5% of pathologically confirmed cases, followed by the face at 26.4% and the nose at 13.9%. These findings highlight the high prevalence of BCC in areas exposed to ultraviolet (UV) radiation, underscoring the critical role of sun exposure in the development of this skin cancer (see Figure 1a,b). Additionally, the localization of lesions in relation to clinical suspicion is illustrated in Figure 2.

### 3.2 Pathological Confirmation

Upon pathological evaluation, 184 cases (80.3%) confirmed the diagnosis of BCC, while 45 patients, who were pathologically confirmed as BCC, received alternative preliminary diagnoses (19.7%). Among these non‐BCC cases, the most frequent conditions included:

- Keratosis (26.7%): A proliferative skin condition that can mimic BCC, potentially complicating clinical diagnostics.
- Inflammatory lesions (20%): This category includes dermatitis, lichen planus, folliculitis, and granuloma annulare. These conditions may present similarly to BCC and require careful differentiation from malignancies.
- Lentigo (9%): A benign skin lesion that may also be mistaken for BCC in cases of hyperpigmentation. The distribution of BCC subtypes among the confirmed cases was as follows (Table 2):
- Nodular BCC: 24.9%.
- Micronodular BCC: 4.4%.

**TABLE 2:** The frequency of each BCC subtype.

Subtype	Frequency
Nodular	57 (24.9%)
Micronodular	10 (4.4%)
Metatypical	8 (3.5%)
Multifocal superficial	8 (3.5%)
Superficial	7 (3.1%)
Infiltrating	5 (2.2%)
Adenoid	4 (1.7%)
Adenoid and nodular	3 (1.3%)
Pigmented	3 (1.3%)
Infundibulocystic	1 (0.4%)
Unknown	123 (53.7%)
Total number of BCC	229 (100%)

### 3.3 Clinical Diagnosis and Referral Sources

Among the 98 patients (42.79%) without a documented clinical diagnosis, 13 cases (13.26%) were referred by dermatologists, while a substantial 84 patients (86.74%) were referred by nondermatologists. The analysis of referral patterns revealed that the surgical ward had the highest referral frequency, at approximately 33%, while the ophthalmology ward had the lowest, at about 10% (see Tables 3 and 4).

**TABLE 3:** Frequency of patients without clinical diagnosis based on referral service.

Referral service	Frequency	Percent (%)
Dermatologist	13	13.26
Ophthalmologist	10	10.20
ENT specialist	16	16.32
Expert surgeon	32	32.65
Internal medicine specialist, gynecologist, unspecified service	27	27.55
Total	98	100

**TABLE 4:** Diagnostic matching based on referral service.

Referral service	BCC suspicion		Total	*p*
	No suspicion	Suspected		
Pediatrician	2	5	7	> 0.05
Dermatologist	4	80	84	
Gynecologist	1	3	4	
Ophthalmologist	0	3	3	
ENT specialist	0	17	17	
Internal medicine specialist	0	3	3	
Anesthesiologist	0	1	1	
Urologist	0	3	3	
Expert surgeon	0	9	9	
Total	7	124	131	

Among the 131 patients with a clinical diagnosis, it was noted that 7 patients (5.4%) did not have documented clinical suspicion. The majority, 124 patients (94.6%), had clinical suspicion recorded. Of these, 102 cases (77.9%) were classified with clinical suspicion as the first differential diagnosis. The remaining cases were documented as follows:

- Second differential diagnosis: 15 patients (11.5%)
- Third differential diagnosis: 6 patients (4.6%)
- Fourth differential diagnosis: 1 case (0.8%)
- In one instance, the order of diagnosis was not specified.

Of the 7 patients lacking clinical suspicion, their referral contexts included:

- Two patients from the pediatric referral service.
- Four patients from the skin referral service.
- One patient from the women's referral service.

It is noteworthy that all patients referred from other services exhibited clinical suspicion, indicating a correlation between service type and clinical suspicion documentation.

### 3.4 Statistical Analysis

Statistical analysis revealed no significant relationship (*p* > 0.05) between the presence or absence of clinical suspicion and the referral source. This suggests that clinical suspicion may not be significantly influenced by the type of healthcare provider referring the patient. It is important to mention that 128 out of the 131 patients with a recorded clinical diagnosis had specific lesion locations noted on their pathology sheets (Table 5), underscoring the detailed tracking of tumor sites which is crucial for future management and study.

**TABLE 5:** Percentage of clinical diagnosis with pathology according to BCC subtype.

Subtype	Total *N*	Concordance (%)
Unknown	54	94.4
Superficial multifocal	8	87.5
Infiltrating	4	100
Micronodular	8	100
Macronodular	1	100
Nodular	40	95
Superficial	8	100
Pigmented	2	50
Metatypical	4	100
Infundibulocystic	1	100
Adenoid	1	100
Total	131	94.6

## 4 Discussion

BCC is a primary malignant epithelial tumor known for its slow progression and ability to invade surrounding tissues [17]. Early diagnosis and a high clinical suspicion are crucial for preventing the progression of advanced tumors [18]. Notably, a significant percentage of patients were diagnosed through referrals from nondermatologists, which highlights the need for enhanced education and training to help recognize skin lesions indicative of BCC.

Our research revealed that only 57 cases (24.9%) involved nodular subtypes, indicating a limited representation of other subtypes in our dataset. The predominance of dermatologists among reporting clinicians likely contributed to the high overall diagnostic accuracy. Additionally, we found no significant differences in clinical suspicion among various BCC subtypes or in diagnostic matching between nodular and nonnodular types or superficial and nonsuperficial types (Table 6; Fisher's Exact test *p* value > 0.05). This uniformity suggests that dermatologist expertise may facilitate accurate diagnosis regardless of the subtype.

**TABLE 6:** The association of lesion subtype with clinical BCC suspicion.

Subtype	With clinical suspicion	Without clinical suspicion	*p* *
Nodular lesion	47	2	0.619
Nonnodular lesion	26	2	
Superficial lesion	15	1	1.00
Nonsuperficial lesion	58	3	
Total	146	8	

* Fisher's exact test was performed.

A critical finding of this study was that only 131 out of 229 pathology reports included a differential clinical diagnosis. Consequently, 98 patients (42%) lacked a clinical diagnosis, which could compromise diagnostic accuracy for pathologists; the absence of a clinical diagnosis may impede informed evaluations. Excluding these cases could potentially lower the clinical agreement rate below the reported 94.6%, aligning it more closely with findings from other studies that have identified similar documentation gaps [19, 20]. Most patients without a clinical diagnosis were referred from nondermatology services, which typically show lower diagnostic accuracy for BCC, emphasizing the need for better training for nonspecialist healthcare providers in recognizing skin cancer symptoms. Previous research supports the need for increased training for nondermatologists to improve early detection of skin malignancies [21].

While many BCC cases are diagnosed and managed without histopathological confirmation, further comprehensive statistical studies are necessary to solidify these findings. Our study, notable for its substantial sample size and retrospective evaluation of clinical versus pathological diagnoses, demonstrated a high concordance rate between these two forms of diagnosis. Clinical diagnoses showed a 94.6% agreement with pathology findings, consistent with results from similar studies conducted across various countries [22, 23, 24]. This high level of agreement may reflect the thoroughness with which physicians conduct differential diagnoses, underscoring the importance of integrating clinical assessments with pathological findings.

Factors such as age, sex, radiation history, and lesion location did not significantly influence diagnostic accuracy in our sample, corroborating previous literature that found minimal impact of demographic variables on clinical recognition of BCC [23, 24]. The nodular subtype constituted the majority of cases, and we found no significant differences in diagnostic compliance between nodular and other BCC subtypes or between superficial and nonsuperficial types (*p* values 0.619 and 1.000, respectively). The lack of significant differences may be due to the smaller statistical representation of nonnodular subtypes in our sample.

BCC mimics, such as squamous cell carcinoma (SCC), nevi, and melanoma, are relevant in both populations, while actinic keratosis and keratoacanthoma are less frequent in individuals with darker skin. Dermoscopy plays a vital role in distinguishing BCC from its mimickers, utilizing patterns like the blue‐white veil and arborizing vessels. However, new features observed in patients with skin of color (SoC) highlight the need for tailored diagnostic strategies [25]. Traditional dermoscopy algorithms may not be suitable for SoC due to differing lesion characteristics, emphasizing the need for further research to develop universally applicable diagnostic tools [25].

In conclusion, our findings advocate for the necessity of clinical diagnosis in managing BCC. It is imperative that all healthcare practitioners involved in the care of BCC patients provide a clinical diagnosis to facilitate the identification of previously undiagnosed cases. Notably, we found no significant relationship between clinical suspicion and the referring service (*p* > 0.05), suggesting that the expertise of individual practitioners may play a more critical role than the type of healthcare service provided. Given that dermatologists excel in diagnosing BCC with high sensitivity and specificity, further extensive studies are warranted to refine these findings and enhance diagnostic strategies across various specialties.

### 4.1 Limitations

One of the limitations of this study is the pathologist's failure to report the exact subtype of BCC in half of the cases, making it challenging to accurately detail the variety of subtypes.

## 5 Conclusion

The clinical diagnosis of BCC is highly accurate among physicians in academic centers, with demographic factors showing no significant impact on diagnostic capability. Notably, 42% of cases lack a clinical diagnosis in pathology applications. Dermatologists often rely on their clinical skills and expertise for accurate diagnosis, making their clinical suspicion the most crucial diagnostic reference. Timely and accurate clinical diagnoses by dermatologists can help reduce complications associated with the disease. Therefore, it is recommended that physicians involved in managing nonmelanoma skin cancers provide clinical diagnoses to enhance the identification of undiagnosed cases and consult specialists, particularly dermatologists, in medical centers.

## Author Contributions

**Fatemeh Farshad:** investigation, writing – original draft. **Elham Behrangi:** conceptualization, methodology. **Alireza Jafarzadeh:** validation, visualization, writing – review and editing. **Masoumeh Roohaninasab:** software, formal analysis, writing – review and editing. **Nasrin Shayanfar:** data curation, resources. **Zeinab Aryanian:** writing – original draft, writing – review and editing. **Parvaneh Hatami:** methodology. **Azadeh Goodarzi:** supervision, conceptualization, project administration.

## Ethics Statement

This study was approved by Ethics Committee with Code no: IR.IUMS.FMD.REC.1397.249.

## Consent

All patients' information remained confidential. All patients signed informed consent for participating in the study.

## Conflicts of Interest

The authors declare no conflicts of interest.

## Data Availability

The data that support the findings of this study are available from the corresponding author upon reasonable request.
